# The impact of obesity on health-related quality of life in Spain

**DOI:** 10.1186/s12955-017-0773-y

**Published:** 2017-10-10

**Authors:** Rafael Busutil, Olga Espallardo, Antonio Torres, Lucía Martínez-Galdeano, Néboa Zozaya, Álvaro Hidalgo-Vega

**Affiliations:** 1 Seminario de Investigación en Economía y Salud, University of Castilla-La Mancha, Toledo, Spain; 2 Hospital Clínico San Carlos, Madrid, Spain; 3 Instituto Max Weber, c/ Norias 123, 28221 Majadahonda, Madrid Spain; 4 University of Castilla-La Mancha, Toledo, Spain

**Keywords:** BMI, Obesity, Quality of life, HRQOL, Spain

## Abstract

**Background:**

It is well documented that obesity is strongly associated with mortality and morbidity, but less is known about its impact on functional status and health-related quality of life (HRQOL). The purpose of this study was to calculate the impact of the Body Mass Index (BMI) on the HRQOL of the Spanish adult population, with special emphasis on BMI ≥ 35.

**Methods:**

We used the Spanish National Health Survey (SNHS) 2011–2012 to assess the statistical association between HRQOL, measured through the EuroQol-5D-5L questionnaire, and the BMI. We conducted linear regression analysis for the EuroQol-5D-5L Visual Analogue Scale (VAS) and probit regressions for each of the five dimensions of the EuroQol-5D-5L.

**Results:**

Self-perceived problems in the five dimensions of the EuroQol-5D-5L increased along the BMI, especially in the mobility and pain/discomfort dimensions. Having a BMI ≥ 35 reduced HRQOL even in the absence of chronic diseases. After controlling for comorbidities, severe obesity decreased the VAS score by an average of 1.9 points and increased the probability of reporting any HRQOL problem in mobility (11.8%), self-care (2.2%), usual activities (4.3%) and pain/discomfort (7.4%). No association was found between obesity and mental problems. All the parameters analysed suggest that HRQOL in women and people aged 65 years and over was significantly worse than average.

**Conclusions:**

BMI is an explanatory factor of self-perceived quality of life. Obesity is associated with a worse HRQOL, especially in women and people aged over 64 years. These results may be useful for designing prevention or treatment health policies to target obesity among the Spanish population.

## Background

Obesity has been described as the epidemic of the twenty-first century [1, 2], and has become a major problem of public health due to its high prevalence and impact on morbidity, mortality, quality of life and healthcare expenditure. In Spain, one in two adults aged 25–60 years has a Body Mass Index (BMI) above the recommendation (BMI ≥ 25 kg/m^2^) and 14.5% are obese (BMI ≥ 30 kg/m^2^) [3]. These figures are expected to continue increasing [4, 5].

Comorbidities of obesity, mainly type II diabetes mellitus, cardiometabolic factors, cardiovascular diseases, asthma, certain cancers and musculoskeletal disorders, have been widely documented [6–9]. Obesity and its associated complications produce a significant deterioration in health-related quality of life (HRQOL) [10]. Various studies have demonstrated that an increase in BMI leads to a decrease in HRQOL, especially in regards to physical aspects and pain [11–14], even in the absence of any other chronic disease [15]. In addition, obese people are more likely to suffer from depression [16, 17] and mood disorders [18], and the probability of suffering from these conditions is higher if obesity develops at an early age [19].

Although it is understood that obesity deteriorates people’s HRQOL, few studies have been conducted on this topic in Spain, and published literature mostly focuses on specific communities or subpopulations [20–23]. The purpose of this study was to calculate the impact of BMI on the HRQOL of the adult population of Spain, especially those suffering from severe to morbid obesity (BMI ≥ 35 kg/m^2^).

## Methods

### Sample

Microdata from the Spanish National Health Survey (SNHS), conducted in 2011–2012, were used as the main source of this analysis. SNHS is a longitudinal population-based survey nationally representative, which includes a total of 26,502 interviews with adults and minors [24]. After excluding individuals with no available data to calculate BMI values and those aged under 18 years old, the analysed sample comprised 18,682 adult subjects, that is representative of the adult population of Spain.

### Body mass index

BMI was calculated using data on self-reported weight and height, and individuals were classified according to the BMI cut-off points established by the World Health Organization (WHO) [6]. Additionally, since patients with a BMI greater than 35 and serious coexisting conditions, or that exceeds 40, are potential candidates for bariatric surgery [25], obesity group was divided into two groups. According to this, the sample was classified as follows: underweight (BMI < 18.5), normal weight (18.5 ≤ BMI < 25), overweight (25 ≤ BMI < 30), moderate obesity, named obesity-A for this study (30 ≤ BMI < 35), and severe to morbid obesity, named obesity-B (BMI ≥ 35). Obesity was subdivided in two groups (obesity-A and obesity-B) in order to be able to analyse if there was a gradual effect of obesity on quality of life, maintaining at the same time the statistical representability of the sample.

### Health-related quality of life

The SNHS 2011–2012 includes the EuroQol-5D-5L (EQ-5D-5L) questionnaire [26], a standardised instrument of HRQOL that provides both a descriptive profile (5 dimensions: mobility, self-care, usual activities, pain/discomfort, and anxiety/depression, each with 5 levels of response according to intensity) and a vertical visual analogue scale (VAS) that records the person’s self-perceived state of health. Validated Spanish country-specific tariffs were used to convert each response combination into Quality Adjusted Life Years (QALYs), by assigning them a utility value which ranged between 0 (equivalent to death) and 1 (perfect health) [27]. The VAS scale ranges from 0 to 100, representing the worst and best imaginable health states, respectively.

The SNHS also includes the Goldberg General Health Questionnaire (GHQ-12) [28], which detects the inability to carry out normal functions of a healthy person and the appearance of new and distressing phenomena, allowing us to analyse the mental health of the sample.

### Sociodemographic characteristics and comorbidities

We included in the analysis those sociodemographic variables (age, gender, nationality, social class, marital status and region of residence [24]), self-reported lifestyle habits, and diagnosed chronic conditions (comorbidities) included in the SNHS that, according to the literature, might be associated with a higher BMI [3, 6–8, 29]. Comorbidities include respiratory diseases (asthma, chronic bronchitis, and COPD), cardiovascular diseases (heart attack, embolism and other heart diseases), muscular diseases (osteoarthritis and lower or upper back pain), stomach diseases (stomach ulcer), diabetes, anxiety and chronic depression, other mental diseases, tumours, migraines, problems with the prostate, thyroid or skin, allergy, urinary incontinence, cataracts, cirrhosis and risk factors (cholesterol and high blood pressure).

### Statistical analysis

Overall, 42 subjects who reported VAS values that contradicted utility values in the EQ-5D-5L were excluded from the analysis [utility > 0.8 and VAS < 20 (*n* = 29); utility <0.2 and VAS > 80 (*n* = 13)].

Linear regression models (Ordinary Least Squares) were used to study the association between VAS values and obesity. Following a progressive inclusion rationale, we ran four different models, all of them controlled by age group and gender: Model 1 additionally controlled for socioeconomic characteristics and BMI; Model 2 controlled for socioeconomic variables, BMI, lifestyle and region of residence; Model 3 controlled for socioeconomic variables, BMI and diagnosed chronic conditions associated with obesity; and Model 4 controlled for socioeconomic variables, BMI, diagnosed chronic conditions, lifestyle and region of residence. Robust unstandardized regression coefficients were estimated.

Additionally, five independent probit regression models were performed, one for each of the 5 health dimensions of the EQ-5D-5L, in order to analyse the impact of obesity on mobility, self-care, usual activities, pain/discomfort and anxiety/depression. The purpose of the probit models is to estimate the probability that an observation with particular characteristics will fall into a specific category. The dependent variables took a value of ‘1’ if any problem was reported in that particular dimension, and ‘0’ if no problem was reported. We also tested the effects by age group (<65 and ≥65 years of age) and gender in separate models.

All explanatory variables took a categorical form (dummy), using as many values as the number of categories in each variable minus one (which would be the reference or comparison category). For instance, BMI was included in the models as four explanatory variables, with “normal weight” being the reference category.

A separate probit regression model was also performed to test the potential relationship between mental health and the BMI, controlling for covariates. GHQ-12 was the dependent variable, taking a value of ‘1’ if any mental problem was reported and ‘0’ if no mental problem was reported.

Statistical analysis was performed using SPSS 22 and Stata 11.0 software. A *p*-value of <0.05 was deemed significant.

## Results

More than half of the Spanish adult population had a BMI that exceeded the WHO recommendation: 37.3% were overweight, 13.3% were moderately obese (obesity-A) and 3.9% were severely to morbidly obese (obesity-B).

**Fig. 1:**
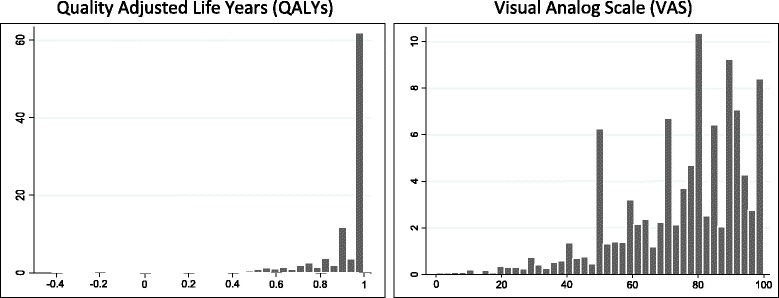
Distribution histograms of QALYs and VAS scores derived from the SNHS

The distribution of the QALYs and the VAS scores resultant from the EQ-5D-5L are shown in Fig. 1. The QALYs’ histogram presents a J-shaped form, as 61.9% of the individuals in the sample reported not having any kind of problems in any of the five dimensions (1 QALY per year assuming that their utility remained constant during the whole year). In contrast, the VAS scores showed a more homogeneous distribution, with only 6.4% reporting a score of 100, and 22.5% reporting a score of 90 or more.

People with normal weight reported a better self-perceived HRQOL than the rest of the sample (Table 1). HRQOL decreased as BMI increased, with obesity-B detracting 0.133 yearly QALYs to normal weight. Mean QALY scores for normal weight people stood for 0.9436, while these figures decreased to 0.9249 among people with overweight, 0.873 for obesity-A and 0.810 for obesity-B.

**Table 1:** Quality-adjusted life years (QALYs) per year, by sex and age group, according to BMI

	Underweight	Normal weight	Overweight	Obesity-A	Obesity-B
Total	0.923	0.943	0.925	0.873	0.810
Sex					
Man	0.893	0.956	0.947	0.911	0.893
Woman	0.932	0.934	0.890	0.825	0.749
Age (years)					
≤ 44	0.966	0.972	0.972	0.951	0.926
45–64	0.866	0.926	0.931	0.902	0.826
≥ 65	0.640	0.827	0.831	0.743	0.632

There were some gender differences. Women reported a lower HRQOL than men in all BMI categories, except for underweight. Obesity seemed to have a greater effect on HRQOL among women. On average, in comparison with respective normal weight groups, women with obesity-B lost 0.185 QALYs per year versus 0.063 for men. QALYs also decreased as age increased, especially with higher BMIs: compared to normal weighted people, those with obesity-B aged under 44 years lost 0.046 QALYs per year, but the loss is 0.100 and 0.195 QALYs for people aged 45–64 years and 65 years and over, respectively.

**Fig. 2:**
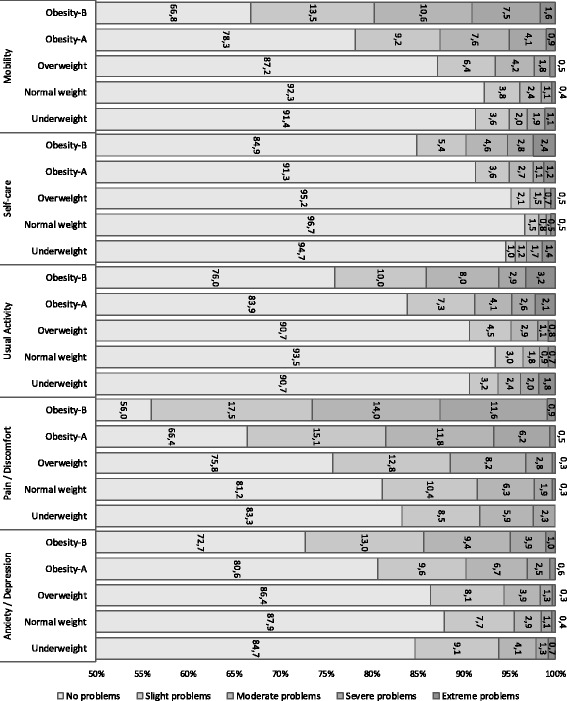
Prevalence of self-reported problems at the five dimensions of the EQ-5D-5L, according to BMI

The analysis of the dimensions of the EQ-5D-5L showed that the frequency of reporting problems in all five dimensions increased with BMI. Overall, 92.3% of respondents with normal weight reported no problems with mobility, while this percentage dropped by 25.5 points in respondents with BMI ≥ 35. This trend was maintained in the rest of the dimensions (Fig. 2). The largest proportion of problems was found in the pain/discomfort dimension, where only 56% of subjects with obesity-B reported having no pain and 12.5% reported severe or extreme pain.

Problems in all dimensions increased in the subsample of respondents aged 65 years and over. Among these subjects, obesity-B increased mobility problems by 36.3%, self-care problems by 20.2%, usual activities by 28.4% and pain/discomfort by 31.4% compared to normal weight peers.

### Regression models

The results from the linear regression models showed that BMI is an explanatory factor of the self-perceived state of health (VAS score), independently of the variables used as control (Table 2). Compared to normal weight, a higher BMI progressively worsened VAS scores. The reductive effects of BMI on VAS got smaller when controlling by chronic diseases (models 3 and 4), as would be expected. In model 4, compared to normal weight, obesity-A and obesity-B reduced the VAS score by an average of 1.9 points (*p* < 0.001) and 3.7 points (*p* < 0.001), respectively. These effects more than double in the simplified models (model 1 and 2), and were also statistically significant. According to the adjusted R-squared values derived from the prediction models, the independent variables explained up to 34% of the total variability of self-perceived quality of life, indicating a fairly good fit.

**Table 2:** Results from the four linear regression models (unstandardized regression coefficients estimated by Ordinary Least Squares)

	Dependent variable: Visual Analog Scale (VAS) of the EQ-5D-5L (score 0–100)			
	Model 1	Model 2	Model 3	Model 4
Man	2.67***	2.40***	0.53*	0.37
45–64 years old	−7.40***	−7.26***	−3.89***	−3.79***
≥65 years old	−14.84***	−15.47***	−7.26***	−7.69***
Upper class	2.55***	2.32***	1.57***	1.40***
Lower class	−1.45***	−1.38***	−0.78*	−0.78*
Foreign	3.60***	3.30***	2.04***	1.83***
Married	−0.44	−0.29	−0.03	0.08
Separated	−2.12***	−1.95***	−0.65	−0.50
Widowed	−4.54***	−4.67***	−2.02***	−2.12***
Underweight	−2.20*	−2.13*	−2.62**	−2.55**
Overweight	−1.83***	−1.67***	−0.72**	−0.61*
Obesity-A	−4.82***	−4.57***	−2.06***	−1.92***
Obesity-B	−9.30***	−8.91***	−3.92***	−3.70***
Northern region		−0.31		−0.35
Southern region		1.06		0.62
Western region		1.71***		1.17***
Eastern region		−0.65		−0.46
Smoking		−1.29***		−0.85**
Alcohol consumption		−4.70***		−4.13***
Physical exercise		−0.47		−0.75*
Respiratory diseases			−3.65***	−3.58***
Vascular diseases			−7.82***	−7.76***
Muscular diseases			−7.34***	−7.35***
Stomach diseases			−3.57***	−3.49***
Diabetes			−4.67***	−4.73***
Depression/anxiety			−9.53***	−9.32***
Risk factors			−2.52***	−2.53***
Other diseases			−3.94***	−3.97***
Constant	83.55***	83.96***	87.90***	88.41***
n	18,516	18,516	18,332	18,332
R^2^	0.189	0.197	0.339	0.344
Adjusted R^2^	0.1884	0.1960	0.3381	0.3432

Model 1: Socioeconomic variables + BMI; Model 2: Socioeconomic variables + BMI + Lifestyle + Region of residence; Model 3: Socioeconomic variables + BMI + Chronic diseases; Model 4: Socioeconomic variables + BMI + Chronic diseases + Lifestyle + Region of residence. The models include robust standardized errors

* *p* < 0.05, ** *p* < 0.01, *** *p* < 0.001

The reference values were the following: woman (for gender), aged under 45 years old (for age group), middle class (for social class), Spanish (for nationality), single (for marital status), no diagnosed chronic disease (for each comorbidity), no smoker, no risky drinker, sedentarism (for lifestyle habits), normal weight (for BMI) and central region (for region of residence)

Description of the independent variables: Social class (Upper: university graduate or director/manager of company; Middle: self-employed person or skilled worker; Lower: semi-skilled or unskilled worker or primary sector worker). Region of residence (Central region: the Community of Madrid, Castilla-La Mancha and Castilla León; Northern region: Navarre, the Basque Country, Aragon and la Rioja; Southern region: Andalusia, Extremadura, Murcia, the Canary Islands, Ceuta and Melilla; Western region: Galicia, Asturias and Cantabria; Eastern region: Catalonia, the Valencian Community and the Balearic Islands). Lifestyle habits (smoking dairy or occasionally; being a risky drinker: drinking more than 35 alcohol units during the past week, if male, or more than 25 alcohol units, if female; physical exercise: walking at least 20 min in the past week). Diagnosed diseases (Respiratory diseases: asthma, chronic bronchitis and COPD; Cardiovascular diseases: heart attack, embolism and other heart diseases; Muscular diseases: osteoarthritis and lower or upper back pain; Stomach diseases: stomach ulcer; diabetes; depression/anxiety: anxiety and chronic depression; risk factors: cholesterol and high blood pressure; other diseases: other mental diseases, tumours, migraines, problems with the prostate, thyroid or skin, allergy, urinary incontinence, cataracts and cirrhosis)

The results of the probit models for each of the EQ-5D-5L dimensions showed how BMI affects each dimension differently (Table 3). Compared to normal weight, obesity-B significantly affects mobility, self-care, usual activities and pain/discomfort, increasing the probability of reporting problems at these dimensions by 11.8%, 2.2%, 4.3% and 7.4%, respectively. However, obesity-B did not have a significant effect on the likelihood of reporting depression/anxiety problems.

**Table 3:** Results from the probit regression models (marginal effects) for each of the EQ-5D-5L dimensions for the sample as a whole

	DIM1: Mobility	DIM2: Self-care	DIM3: Usual Activities	DIM4: Pain/Discomfort	DIM5: Anxiety/Depression
Man	−0.0007	−0.0023	−0.0063	−0.0609**	−0.0301**
45–64 years old	0.0748**	0.0219**	0.0351**	0.0559**	0.0364**
≥65 years old	0.2350**	0.0855**	0.1196**	0.1203**	0.0240*
Upper class	−0.0246**	−0.0079*	−0.0167**	−0.0453**	−0.0076
Lower class	0.0065	0.0076*	0.0117*	0.0168	0.0212*
Foreign	−0.0078	−0.0063	−0.0161*	0.0002	0.0267*
Married	−0.0158*	−0.0052	−0.0056	0.0063	−0.0061
Separated	−0.0139	0.0020	−0.0007	−0.0001	0.0200
Widowed	0.0259*	0.0149*	0.0311**	0.0329*	0.0403*
Respiratory diseases	0.0314**	0.0057	0.0255**	0.0539**	0.0169
Vascular diseases	0.0924**	0.0398**	0.0856**	0.1010**	0.0523**
Muscular diseases	0.1109**	0.0334**	0.0815**	0.3027**	0.0481**
Stomach diseases	0.0272*	0.0017	0.0065	0.0532*	0.0224
Diabetes	0.0602**	0.0170**	0.0413**	0.0549**	0.0122
Depression/anxiety	0.0701**	0.0253**	0.0764**	0.1522**	0.5364**
Risk factors	0.0032	−0.0005	0.0022	0.0461**	0.0247**
Other diseases	0.0472**	0.0188**	0.0509**	0.0779**	0.0301**
Smoking	0.0071	−0.0028	−0.0001	0.0024	0.0261**
Alcohol consumption	0.0045	−0.0118*	−0.0120	0.0156	−0.0086
Physical exercise	−0.0088	0.0002	−0.0114*	−0.0150*	−0.0123*
Underweight	0.0289	0.0326*	0.0501*	0.0297	0.0383
Overweight	0.0066	−0.0040	−0.0051	0.0041	−0.0068
Obesity-A	0.0334**	0.0013	0.0101	0.0366*	0.0085
Obesity-B	0.1182**	0.0217*	0.0427**	0.0743**	0.0199
Northern region	−0.0172*	0.0004	0.0128*	−0.0319*	−0.0134
Southern region	0.0239**	0.0127**	0.0337**	−0.0017	0.0105
Western region	0.0172*	0.0111*	0.0304**	−0.0182	0.0190*
Eastern region	0.0141*	0.0059	0.0278**	0.0262*	0.0338**
n	18,444	18,444	18,443	18,438	18,433
Pseudo R^2^	0.319	0.284	0.301	0.259	0.309

Probabilities of developing a problem

* *p* < 0.05; ** *p* < 0.001

The reference values were the following: woman (for gender), aged under 45 years old (for age group), middle class (for social class), Spanish (for nationality), single (for marital status), no diagnosed chronic disease (for each comorbidity), no smoker, no risky drinker, sedentarism (for lifestyle habits), normal weight (for BMI) and central region (for region of residence)

Description of the independent variables: Social class (Upper: university graduate or director/manager of company; Middle: self-employed person or skilled worker; Lower: semi-skilled or unskilled worker or primary sector worker). Diagnosed diseases (Respiratory diseases: asthma, chronic bronchitis and COPD; Cardiovascular diseases: heart attack, embolism and other heart diseases; Muscular diseases: osteoarthritis and lower or upper back pain; Stomach diseases: stomach ulcer; diabetes; depression/anxiety: anxiety and chronic depression; risk factors: cholesterol and high blood pressure; other diseases: other mental diseases, tumours, migraines, problems with the prostate, thyroid or skin, allergy, urinary incontinence, cataracts and cirrhosis). Lifestyle habits (smoking dairy or occasionally; being a risky drinker: drinking more than 35 alcohol units during the past week, if male, or more than 25 alcohol units, if female; physical exercise: walking at least 20 min in the past week). Region of residence (Central region: the Community of Madrid, Castilla-La Mancha and Castilla León; Northern region: Navarre, the Basque Country, Aragon and la Rioja; Southern region: Andalusia, Extremadura, Murcia, the Canary Islands, Ceuta and Melilla; Western region: Galicia, Asturias and Cantabria; Eastern region: Catalonia, the Valencian Community and the Balearic Islands)

Table 4 reveals the results after distinguishing by age and gender. In the full sample, mobility was the dimension most affected by obesity, followed by pain/discomfort. When separated models by age groups were analyzed, the probability of reporting any problem at any EQ-5D-5L dimension was significantly higher in the obese ≥65 age group (both genders) when compared to normal weight old people. In this group, the probability of reporting any mobility problems increased from 3.3% to 9.5% with obesity-A and from 11.8% to 18% with obesity-B. In people under 65, the associated probabilities drop to 2% and 9.3%, respectively.

**Table 4:** Results from the probit regression models (marginal effects for obesity-A and obesity-B) for each of the EQ-5D-5L dimensions by sex and age group: probability of developing a problem in each dimension

	Dim 1: Mobility			Dim 2: Self-care			Dim 3: Usual Activity			Dim 4: Pain/Discomfort			Dim 5: Anxiety/Depression		
All ages															
	Both sexes	Men	Women	Both sexes	Men	Women	Both sexes	Men	Women	Both sexes	Men	Women	Both sexes	Men	Women
Obesity-A	0.0334	–	0.0502	–	–	–	–	–	0.0188	0.0366	–	0.0575	–	–	–
Obesity -B	0.1182	0.0640	0.1623	0.0217	–	0.0414	0.0427	–	0.0760	0.0754	–	0.1288	–	–	–
Pseudo R^2^	0.319	0.278	0.341	0.284	0.250	0.293	0.301	0.258	0.318	0.259	0.207	0.271	0.309	0.252	0.320
Less than 65 years old															
	Both sexes	Men	Women	Both sexes	Men	Women	Both sexes	Men	Women	Both sexes	Men	Women	Both sexes	Men	Women
Obesity-A	0.0202	–	0.0290	–	–	–	–	–	–	–	–	0.0560	–	–	0.0405
Obesity-B	0.0932	0.0579	0.1212	0.0145	–	0.0225	0.0204	–	0.0365	0.0542	–	0.1132	–	–	–
Pseudo R^2^	0.217	0.205	0.238	0.199	0.186	0.213	0.245	0.238	0.257	0.216	0.177	0.234	0.289	0.253	0.302
65 years old and over															
	Both sexes	Men	Women	Both sexes	Men	Women	Both sexes	Men	Women	Both sexes	Men	Women	Both sexes	Men	Women
Obesity-A	0.0953	–	0.1203	–	–	–	0.0439	–	0.0547	–	–	–	–	–	–
Obesity-B	0.1800	–	0.2328	0.0670	–	0.1243	0.1350	–	0.1970	0.0931	–	0.1372	–	−0.0537	–
Pseudo R^2^	0.178	0.160	0.180	0.158	0.159	0.150	0.190	0.171	0.185	0.200	0.165	0.198	0.322	0.261	0.324

Note: only statistically significant marginal effects are shown (*p* < 0.05), otherwise “–” is shown

The marginal effects for obesity-A and obesity-B for the sample as a whole (coloured areas) correspond to the effects showed in Table 3

The results by gender show that the only dimension that is significantly likely to be affected by obesity in men is mobility, both in the sample comprising “all ages” and in the sample of younger men. By contrast, women of both age groups with obesity-B were more likely to report problems in all dimensions but anxiety/depression, compared to women with normal weight. Regardless of their age, obesity-B in women increased by 16% the probability of declaring mobility problems compared to 6% in men. This probability rose to 23% in women aged over 65 years. In women under 65, obesity-B increased the probability of reporting problems related to mobility, self-care, usual activities and pain by 12.1%, 2.3%, 3.7% and 11.3%, respectively, compared to women with normal weight. These values rose to 23.3%, 12.4%, 19.7% and 13.7%, respectively, for women aged 65 years or over. The pseudo R-squared values derived from the probit models indicated a fairly goodness-of-fit, with better predictions of the outcome for the general population than for subsets.

The results of the model using the GHQ-12 scores as the dependent value (not reported in tables) were consistent with the results obtained for the depression/anxiety dimension of the EQ-5D-5L. There was no statistically significant association between BMI and mental health (*p* = 0.606 for obesity-A and *p* = 0.842 for obesity-B).

## Discussion

Obesity is related to the development of comorbidities that impair not only the person’s objective health status but also their self-perceived health. In this study we have confirmed, through different econometric models, that obesity is a determining factor of a worse HRQOL, whether the latter is measured using a linear thermometer (VAS scores) or through the reporting of problems in the five dimensions of the EQ-5D-5L. The EQ-5D is a generic instrument (the only HRQOL instrument included in the SNHS) for measuring HRQOL, that is widely used for economic evaluation in many areas of health research.

Both the Visual Analogue Scale and the EQ-5D-5L descriptive system conform the EuroQol, but they measure conceptually different aspects of self-perceived health. They are highly correlated (Spearman test coefficient: 0.587; *p* = 0.00), but their distributions showed substantial differences (Fig. 1), which could be partially attributed to the following factors. The ‘end-point aversion’ is a measurement bias that occurs when people are not likely to use the extreme ends of the scales [30]. Health states perceived as worse than death do not have a proper representation on the VAS, unlike the EQ-5D-5L index, which allows for disutilities in extremely severe health states. Besides, as a direct measure of self-perceived health status, the VAS is more subject to the patient’s interpretation and subjectivity than QALY scores. We tried to control this problem by dismissing outliers from the analysis.

To our knowledge, this is the first study that analyses the effects of BMI on HRQOL using the EQ-5D-5L questionnaire, which is more precise than its previous version (EQ-5D-3L) comprising only three possible problem level answers.

Our results show that the greater the excessive BMI, the higher the probability of having a low self-perceived health status, regardless of age and gender. According to our findings, moderate obesity and severe-to-morbid obesity reduced self-perceived health status by approximately 2 and 4 points, respectively, after controlling for diagnosed chronic diseases.

With respect to the EQ-5D-5L dimensions, obesity-B primarily affected mobility and pain/discomfort, and to a lesser extent usual activities and self-care. However, obesity did not appear to be associated with depression or anxiety problems. This is consistent with the analysis conducted on the GHQ-12, that suggested that there was no significant association between obesity and mental health.

Women systematically reported a lower HRQOL than men. This trend was exacerbated by the deeper negative effect of obesity on women’s self-perceived quality of life. Among people aged 65 years and over, obesity-B was statistically associated with lower HRQOL. This association was stronger than that in people under 65 years. Women were particularly affected by this age effect.

Our results are consistent with the published empirical literature. Among other studies conducted in Spain, Serrano-Aguilar et al. found that obesity in the Canary Islands had a significant negative impact on HRQOL, even in people who did not suffer from chronic diseases. The authors showed that people with BMI ≥ 25 had a higher probability of indicating a worse HRQOL, and that the effect was greater in women than in men. People with class II obesity (35 ≤ BMI < 40) had a 47% higher probability of reporting a worse HRQOL, while people with class III obesity (BMI ≥ 40) were 3 times more likely to have a worse HRQOL than people with normal weight [22]. Similarly, Oliva-Moreno et al. found that the effects of severe to morbid obesity were greater than those of moderate obesity, that excess weight significantly diminished self-perceived health status, and that this effect was stronger in women than in men. In their study, where the EuroQol questionnaire with 3 levels was used, the most affected dimension by obesity was also mobility, followed by pain/discomfort, in line with our results [23].

At worldwide level, some studies have corroborated the significant and negative association between BMI and self-perceived quality of life, measuring the latter with different instruments such as the EQ-5D-3L or the Short-Form 36 (SF-36) [9, 11–15, 31–36]. Most of these studies confirmed that the malignant effect of obesity is greater in women than in men and that obesity is linked to a worse physical HRQOL, but there is no consensus on the mental HRQOL component.

It is well stated that women self-report worse HRQOL than men [37]. This gap is largely explained by sociodemographic and socioeconomic status differentials between men and women, but there are other factors that may be relevant, such as differential reporting patterns. In elderly women, the difference seems to be mainly due to a higher prevalence of disability and chronic conditions [38].

While obesity is a well-recognized risk factor for impaired HRQOL, few studies have investigated the underlying biological mechanisms of this relationship. The effects of obesity on obesity-related diseases and self-reported health are expected to be involved in the mechanisms underlying the obesity-HRQOL association. Some authors argued that the long-term physiological effects of obesity are numerous and potentiate each other [39]. Park et al. explored the potential causal pathways and mediating effects of the pathologic conditions that may explain this association and the difference among gender groups [36]. The study concluded that obesity was only directly associated with HRQOL in women. In men, it was indirectly associated with HRQOL through diabetes mellitus, hypertension, dyslipidemia and self-rated health. Psychological differences between genders, such as dissatisfaction with body shapes, weight self-stigmatization, internalization of weight bias and higher perceived health risk associated with obesity could account for this disease-independent pathway.

The relationship between obesity and mental health has been subject of considerable debate. Alike the present study, several reviews and individual studies were inconclusive in relation to the influence of obesity on mental HRQOL, reporting no or very weak association [40–42]. However, a recent meta-analysis concluded that both mental and physical quality of life were impaired in class III obese individuals (BMI ≥ 40), although it founded no statistically significant association for less obese people [34]. Some biological studies indicated that stress, anxiety and depression could be the cause of obesity and that a two-way causal relationship may exist [43]. Poor emotional well-being among the obese may be due to comorbidity rather than obesity per se [44]. Generic measures of HRQOL might not be sensitive enough to reflect impairment in mental HRQOL, especially among those with less severe obesity.

Our results are not free from limitations. First, the SNHS provides self-reported HRQOL measured at a specific moment in time, which does not enable us to analyse the evolution over time or the causality between obesity and lower self-perceived HRQOL. Some longitudinal studies studied the evolution of this relationship over time [45, 46]. Second, in the SNHS, HRQOL was measured through self-reported questionnaires, thus providing a subjective value, dependent on the relative perception of the person at that specific point of time. In addition, BMI is derived from self-reported data of height and weight. If, as previous studies suggested, people tend to underestimate their weight and to overestimate their height, our results may be underestimating the real prevalence of obesity in the sample [17, 47–49]. To conclude, some studies argue that BMI is not a valid measurement of obesity for people older than 65 [50]. Therefore, our results for this populations’ segment should be interpreted with caution.

Our results could serve as a basis for designing and implementing health policies. Over the last decades, interventions to address the growing obesity epidemic have escalated [39]. Available modalities for the treatment of adult obesity include clinical counselling focused on diet and physical activity, pharmacotherapy, and bariatric surgery [51, 52]. Because the prevalence of obesity poses an enormous clinical burden, innovative treatment strategies are needed, whose success will depend on taking into account relevant cultural, economic and social aspects. Decision makers should have access to key sources of data on the burdens associated with obesity, such as reduced life expectancy and lower health-related quality of life, in order to build sound policies focused on reducing the negative effects of obesity on today’s society.

## Conclusions

Overweight and obesity affect more than half of the Spanish adult population. We showed that obesity is associated with a worse health-related quality of life, especially in women and people over the age of 65. A BMI over 35 appears to be prejudicial for mobility and discomfort, affecting daily activities, but we found no connexion between obesity and mental problems such as anxiety or depression. Despite the limitations of our study, we can confirm that the data from the SNHS 2011–2012 provide new and valid information on the impact of obesity on people’s self-perceived HRQOL, as well as on the different ways in which it affects people according to their gender and age group. This information may be useful for designing prevention and treatment health policies that target obesity among the Spanish population.

## Abbreviations

BMI: Body Mass Index
COPD: Chronic Obstructive Pulmonary Disease
EQ-5D-3L: EuroQol-5D-3L
EQ-5D-5L: EuroQol-5D-5L
GHQ-12: Goldberg General Health Questionnaire
HRQOL: Health-Related Quality of Life
QALYs: Quality Adjusted Life Years
SNHS: Spanish National Health Survey
VAS: Visual Analogue Scale
WHO: World Health Organization
